# Ethics of artificial intelligence in prenatal and pediatric genomic medicine

**DOI:** 10.1007/s12687-023-00678-4

**Published:** 2023-10-05

**Authors:** Simon Coghlan, Christopher Gyngell, Danya F Vears

**Affiliations:** 1 School of Computing and Information Systems (CIS), Centre for AI and Digital Ethics (CAIDE), The University of Melbourne, Grattan St, Melbourne, Victoria 3010 Australia; 2 Australian Research Council Centre of Excellence for Automated Decision Making and Society (ADM+S), Melbourne, Victoria Australia; 3 Biomedical Ethics Research Group, Murdoch Children’s Research Institute, The Royal Children’s Hospital, 50 Flemington Rd, Parkville, Victoria 3052 Australia; 4 University of Melbourne, Parkville, Victoria 3052 Australia; 5 Centre for Biomedical Ethics and Law, KU Leuven, Kapucijnenvoer 35, 3000 Leuven, Belgium

**Keywords:** Artificial intelligence (AI), Ethics, Genomics, Pediatrics, Prenatal medicine, Children

## Abstract

This paper examines the ethics of introducing emerging forms of artificial intelligence (AI) into prenatal and pediatric genomic medicine. Application of genomic AI to these early life settings has not received much attention in the ethics literature. We focus on three contexts: (1) prenatal genomic sequencing for possible fetal abnormalities, (2) rapid genomic sequencing for critically ill children, and (3) reanalysis of genomic data obtained from children for diagnostic purposes. The paper identifies and discusses various ethical issues in the possible application of genomic AI in these settings, especially as they relate to concepts of beneficence, nonmaleficence, respect for autonomy, justice, transparency, accountability, privacy, and trust. The examination will inform the ethically sound introduction of genomic AI in early human life.

## Introduction

The movement of artificial intelligence (AI) into medicine is attracting increasing ethical debate and discussion (Keskinbora 2019; Morley et al. 2020; Nucci 2019; Sand et al. 2022). In this paper, we examine ethical questions concerning the introduction of artificial intelligence (AI) into prenatal and pediatric genomic medicine. The likely increasing application of AI to genomic medicine in early human life is yet to receive detailed ethical treatment.^1^ This contrasts with other medical areas such as oncology, psychiatry, ophthalmology, and radiology where the ethics of using AI has already been explored at some length (Carter et al. 2020; Morgan and Mates 2023; Rogers et al. 2021; Shen et al. 2022; Shreve et al. 2022).

AI solves problems in ways analogous (but not equivalent) to humans by inferring, classifying, and predicting (Russell and Norvig 2021). This ability could enable AI to assist in significant medical activities such as diagnosing, prognosing, and making treatment recommendations. Contemporary AI often involves machine learning (ML), which requires large datasets to train algorithms to make predictions (e.g., about medical diagnosis or prognosis) based on generalizations from the data (Greenhill and Edmunds 2020). For example, a model trained on hundreds of radiographic images might “learn” to pinpoint subtle lesions in bones (Park et al. 2022).

ML is suited to detecting, often very rapidly, patterns in data too intricate, multidimensional, and complicated for humans to discern. A well-publicized example of AI’s advances is the ML system AlphaFold, which predicted the folding structure of 600 million proteins from their amino acid sequences alone (Callaway 2022). Like AlphaFold, much ML today involves so-called *deep learning*. Deep learning models feature artificial neural networks which are arrayed in multiple hidden layers and can learn highly complex and non-linear statistical patterns amongst the inputted data (Russell and Norvig 2021). ML models used in medicine may operate on health-related data of various kinds, including electronic health records, images, audio, drug data, electric medical signals, and genetic data—or combinations of such data (Rajpurkar et al. 2022).

Already, AI can sometimes equal or outperform medical experts. For example, a computer vision algorithm trained on expert-labelled pathology slides was better at detecting lymph node metastasis of breast cancer than expert pathologists (Ehteshami Bejnordi et al. 2017), a deep learning model proved equal to board-certified ophthalmologists in diagnosing diabetic retinopathy (Gulshan et al. 2016), and another deep learning algorithm outperformed six radiologists in the task of detecting lung cancer on radiographic images (Ardila et al. 2019).

AI is being researched and developed for medical use in young humans too. Examples of pediatric applications include identifying sepsis, pulmonary hypertension, autism, asthma, and cancer (Sisk et al. 2020) and designing stem cell therapies for children (Sniecinski and Seghatchian 2018). In pediatric genomics, ML can classify diseases by combining pathologic information, genomic information, and medical records and can re-analyze cases where no genetic cause has yet been established (Zou et al. 2019).

AI’s promise in medicine appears to be high (Topol 2019). Yet AI also carries risks and raises ethical questions which are best examined before it becomes more pervasive. This need extends to genomic medicine in early human life. In this paper, we examine the ethics of AI applications in three early human life settings: (1) prenatal genomic sequencing for possible fetal abnormalities, (2) rapid genomic sequencing for critically ill children (e.g., in intensive care units), and (3) reanalysis of genomic data obtained from children for diagnostic purposes. These genomic AI applications raise several moral questions which we suggest will profit from consideration of certain ethical concepts, including concepts of beneficence, nonmaleficence, respect for autonomy, justice, transparency, trust, accountability, and privacy.

The paper runs as follows. We first present some background on genomic AI in early human life. We then draw on AI ethics and medical ethics to identify ethical considerations relevant to possible uses of genomic AI. We next proceed to the main examination of genomic AI in the three target domains, and we finish by discussing when AI might be morally justified. Our analysis can be used to evaluate and inform the ethically sound introduction of genomic AI into prenatal and pediatric settings.

## Genomic AI in prenatal and pediatric settings

Genomic AI technologies could be used in various ways in early human life. For example, ML models trained on millions of DNA samples could assist with variant calling, genome annotation, and variant classification to assist genetic diagnosis in unborn or young humans (Quang et al. 2015). In pediatric populations, ML models trained on both genetic and phenotypic data might also predict genotypes from phenotypes (e.g., in a young patient with certain facial dysmorphologies) or phenotypes from genotypes (e.g., in a fetus with rare pathogenic variants). In one study, automated genetic analysis prospectively diagnosed three out of seven critically ill infants in ICU (Clark et al. 2019). Screening children’s genetic and other data using AI may also improve prediction of future medical conditions.

To be sure, this technology is in its infancy and mature AI systems for pediatric clinical diagnosis and treatment are still being established (Li et al. 2020). Nonetheless, genomic medicine appears ripe for AI interventions in pre/early life. This is partly due to the increasing availability of genomic data (Williams et al. 2018) and the fact that manual interpretation of complex genomic and phenotypic data is labor-intensive and requires considerable expertise. AI might improve efficiency, reduce costs,^2^ and extend access to genomic services. Additionally, such systems may sometimes do better than clinicians in identifying genetic syndromes, including those that are overlooked due to their rarity (Kuru et al. 2014).

## Guiding ethical concepts in medical and AI ethics

To guide our subsequent examination, we highlight several relevant ethical concepts from medical and AI ethics. Sometimes framed as ethical “principles,” these concepts are regarded by at least many scholars as important and widely applicable ethical ideas in medical and/or AI-related activity. Such concepts can help guide reasoning about the ethical duties and responsibilities of health personnel, clinics or hospitals, and AI designers and developers. Here, we identify and briefly explain the relevant guiding ethical concepts.

The ethical principles of beneficence, non-maleficence, respect for autonomy, and justice are commonly (though not unanimously (Rhodes 2020)) adopted principles for ethical reasoning in medicine (Beauchamp and Childress 2001). These principles, or some version of them, may be adapted to AI contexts generally (Floridi et al. 2018) and medical AI contexts specifically (Rogers et al. 2021). For example, use of a new AI system might be partly justified by its potential to promote beneficence by improving clinical diagnosis. At the same time, that system’s undeclared use might be felt to disrespect patient (or guardian) autonomy, at least in cases where the system is relatively untested or where patients (or their guardians) are known to have special concerns about medical AI (Scott et al. 2021).

Again, we might feel that an AI tool accords with a guiding concept of nonmaleficence by, for instance, mitigating the tiredness, distraction, and cognitive bias which cause humans to make medical errors (O’Sullivan and Schofield 2018). An AI tool might also align with a principle of justice by, say, improving access to diagnostic capabilities amongst groups relatively neglected in healthcare (Currie and Hawk 2021). Alternatively, a hyped AI system might conflict with some of these principles when it produces harms without adequate benefits, or generates unjust bias and discrimination against individuals or groups.

Although AI ethics scholars often apply the above ethical principles in some form to AI, they also suggest additional guiding concepts for morally evaluating those technologies (Jobin et al. 2019). Arguably, the ethical concepts of transparency, accountability, privacy, and trust are especially important for medical AI. We discuss each in turn.

The principle of transparency (Floridi et al. 2018) is frequently highlighted by AI ethicists. One kind of transparency concerns disclosure to relevant parties of the nature and usage of impactful AI systems. This could include disclosure to patients or to their parents or guardians. Another kind of transparency concerns proprietary secrecy, where company policies or commercial-in-confidence arrangements prevent health practitioners and patients/guardians from understanding how the AI works and its possible weaknesses.

Transparency is also related to the algorithms themselves. Deep learning models, in contrast to some other ML models like decision trees, are said to lack transparency when they do not afford interpretable accounts of the basis of their predictions and inferences (Xu et al. 2019). In some AI and ML models, the ground of the inference is readily understandable, e.g., “patient probably has pneumonia due to clinical signs x, y, z.” In deep learning models, by contrast, the underlying patterns on which the inferences are based can be too mathematically complicated to be understood (Payrovnaziri et al. 2020). Such models are termed “blackboxes” (Quinn et al. 2021). Research in explainable AI attempts to render blackbox models more intelligible and less opaque by providing sufficiently useful interpretations of their inferences (Gunning et al. 2019).

ML models lacking transparency potentially create or accentuate ethical issues in medicine (and elsewhere) (Kundu 2021). For a start, algorithmic opaqueness can make it harder for personnel to identify errors in prediction or classification, including errors that no human with expertise would ordinarily make. One reason for this is algorithmic brittleness, in which models, despite being accurate much of the time, fail when applied to cases outside their key training set (Cummings 2021). A notorious example from computer vision is where an AI model tagged dark skinned human faces as “gorillas” (Birhane 2022). Sometimes, deep learning models may issue correct outputs based on an erroneous classification which remains hidden in the neural network.

Additionally, non-transparent AI can hide algorithmic biases that arise in ML training or deployment (Caruana et al. 2015). Such biases may be harmful to patients and/or unfair (Quinn et al. 2021). A stark example of algorithmic bias is a medical AI model that recommended less intensive treatment for black compared to white patients (Obermeyer et al. 2019). The bias resulted because the model used healthcare expenditure as a proxy for medical need, and black patients typically have poorer access to health services due historic disadvantage and lower insurance rates. Although Obermeyer et al. (2019) do not identify the precise nature of that ML model, their example is often used to illustrate an acute ethical problem with non-transparent or blackbox AI (Petch et al. 2022).

For such reasons, some AI experts argue that interpretable models should almost always be preferred (Rudin 2019). Others commentators, however, argue that the ethical problems of blackbox AI are overstated (London 2019). For example, a deep learning model may be opaque but nonetheless more accurate than either interpretable models or medical experts. After all, much of medicine, including the exact causal mechanisms of certain conditions and some efficacious treatments, is opaque. Furthermore, AI biases may be predictable or discoverable *ex ante* even in blackbox systems.^3^

Some such biases, however, may go unnoticed even when obvious sources of bias in models are removed or controlled for (Yang et al. 2022). These biases are more difficult to detect in non-transparent AI systems. The same difficulty in detection applies to algorithmic brittleness and error. Erroneous outputs of this sort can increase the risk of harm to some patients, possibly on a significant scale when the AI is widely used. Thus, even if a blackbox model is generally accurate and medically beneficial, it may nevertheless result, in certain cases, in overlooked patient harms and unjust biases. This raises the prospect of having to make ethical tradeoffs (Amann et al. 2020).

The importance of accountability has also been highlighted (Smith 2021). For example, a health practitioner or medical facility that chooses to rely on AI software that is prone to error or bias, or that has been insufficiently tested, presumably ought not pass the ethical buck to the AI developer. There is an ongoing risk here of AI conformity and automation bias—a recognized phenomenon in which humans unconsciously place excessive trust in computers and algorithms (Goddard et al. 2012). That said, if a medical AI tool begins to routinely surpass human accuracy, it may become increasingly debated to what extent practitioners, clinics, or hospitals should be held fully morally (and legally) accountable for undesirable patient outcomes, as opposed to AI developers or vendors (Prictor 2022).

AI scholars have also underlined the ethical notion of privacy (Zhang et al. 2021). Training ML models typically requires copious and sensitive data from patients in the original training of the algorithms and in subsequent refinements. These data are susceptible to misuse, hacking, mishandling, and leakage (Khan et al. 2023). Accordingly, AI ethicists have stressed the need for upholding data security (Jobin et al. 2019) and further research into it.

The final prominent concept in AI ethics relevant to medical AI is trust (Jacovi et al. 2021). Risks of harm, privacy loss, non-transparency, and insufficient accountability can all affect trust in medical AI systems (Durán and Jongsma 2021). On the one hand, unwarranted trust in machines may result in unethical use of AI tools; on the other hand, unwarranted distrust amongst patients, practitioners, and regulators may prevent their beneficial adoption. For some, public and political concern about AI, plus its fast pace and relative novelty, could heighten distrust and simultaneously increase the desirability of transparency and openness in high-stakes uses—at least until medical AI is more widely understood and accepted.

In this section, we outlined ethical considerations that affect many kinds of medical AI uses. Next, we apply the above guiding ethical concepts to genomic AI in prenatal and pediatric settings. We mostly focus on ethical issues somewhat distinctive to these settings, though we sometimes highlight issues that also have more general application in medicine but are still important for those involved in genomic AI to appreciate. Identifying these ethical issues should allow them to be better anticipated and addressed.

## Three settings of genomic AI application in early life

Below we examine AI applied in three settings: prenatal genomic sequencing, rapid genomic sequencing for critically ill children, and reanalysis of genomic data obtained from children. These settings each raise some similar and different issues. We should stress that the precise risks and benefits of genomic AI would need to be established for each application and model, not just for each early life setting. Different AI models can have quite different workings and implications. Furthermore, different contexts (e.g., different patient cohorts) could affect the accuracy and fairness of a given algorithm, as could ongoing changes to the algorithm due to the input of new data.

### AI in prenatal genomic sequencing

One of the most costly and time-consuming aspects of GS is “variant calling.” When a patient’s genome is sequenced, there are likely to be many different genomic variations compared to a reference genome. Most of this variation will have a negligible effect on people’s health. Identifying which variants are part of the natural background variation and which cause disease is thus a major challenge for GS. Clinical interpretation of genetic variants in the context of the patient’s phenotype remains largely manual, is extremely labor-intensive, and requires highly trained expert input.

Use of genomic sequencing (GS) is increasingly prevalent for fetuses identified as having an abnormality on ultrasound. GS has a much greater chance of identifying the likely cause of fetal abnormality than the previous gold standard: chromosomal microarray (CMA) (Dugoff et al. 2016). However, GS also has greater potential to identify incidental findings (Fu et al. 2022; Guadagnolo et al. 2021; Plantinga et al. 2022; Vears et al. 2018) — that is, variants in disease-causing genes that are unrelated to the phenotype under investigation and found by chance during analysis. Incidental findings require decisions about whether they are reported back to the referring clinician and patient or prospective parents. Whether or not to return incidental findings and/or secondary findings in the prenatal setting has been highly contested and few guidelines published by professional bodies address this issue (Vears and Amor 2022). However, decisions made during the analysis also influence whether or not these incidental findings are seen in the first place (Vears et al. 2021).

Laboratory scientists can use bioinformatic filters to “mask” particular sets of genes they wish to exclude from the analysis. Scientists could for example mask *BRCA* genes that predispose to breast and ovarian cancer when they are analyzing the GS data of a child, to preserve their right not to know their at-risk status and so promote their future autonomy as adults. A laboratory with parental samples can also filter by inheritance pattern so that only new (*de novo*) variants in the fetus, or variants requiring two copies of the gene to be knocked out (one from each parent; autosomal recessive conditions), will be seen. Both strategies can minimize incidental findings that could have implications for children, and also for their parents.

For parents who wish to avoid findings that are associated with unclear benefits, these filtering strategies can help promote autonomous choices and avoid causing them harm in the form of anxiety or distress. However, some parents may genuinely want to receive all findings, even if there is only a small chance those findings will be actionable or clinically relevant for their child. For these parents, filtering may subvert rather than promote autonomy.

As we noted, current analysis practices for GS data require laborious manual curation (bringing together and weighing the evidence to decide whether the variant/s identified are the cause of the condition under investigation). Hence, AI for analyzing prenatal GS data could bring significant benefit for patients and society more generally by increasing speed and efficiency and decreasing costs. In the short term, automated analysis and curation of the data would reduce turnaround times for issuing reports, which is particularly important in the time-critical prenatal setting. AI could facilitate incorporation of data from multiple databases that hold the critical information for making judgments about the likelihood a variant is responsible for the abnormalities identified. It could also incorporate an ML system where over time the AI could improve and refine its processes.

Theoretically, ultrasound information would also be used as a filter to ensure genes selected for analysis are consistent with seen abnormalities. ML methods used in ultrasound can find correlations between data that do not necessarily map onto causes of conditions (Dastani and Yazdanpanah 2023). This need not always affect accuracy and utility, though sometimes it will. The better the incorporation of the evidence, such as population variant frequencies, protein modelling, and genotype-phenotype correlations, the more accurate the prediction of variants likely to be causative. As more available genome data are fed through the system, the learning capabilities of the AI system may increase the accuracy of predictions of pathogenicity (although that is certainly not guaranteed).

Perhaps more importantly, as changes in the population occur, artificial learning capabilities that are not “locked” but that rather receive updated training on relevant new data could react more quickly than human personnel, who would themselves need updated training. Equally importantly, AI analysis is less resource-intensive because manual curation requires laboratory scientists, of which there is a shortage.

However, genomic AI in this prenatal domain also raises risks. To start with, imagine a future where, instead of a laboratory scientist making decisions about which genes are analyzed and which findings are returned from prenatal GS, we rely solely on the analysis and outputs of a powerful DNN. Blackboxes lack intrinsic transparency because the input data is analyzed in ways even the programmers do not understand. Accordingly, it may be unclear how decisions about which genes were included in the analysis were made. In some cases, it may be hard to decipher the ruleset used to determine which results get returned to the referring doctor and parents. This is particularly challenging considering the very real potential for incidental findings to be identified.

It is thus necessary to design ML systems from the outset to reduce the chances of returning incidental findings. Even then, a lack of interpretability could diminish autonomy. While in most cases prospective parents may not want to know the underlying reasoning behind a child’s diagnosis, or how reliable a diagnosis is, both are reasonable requests that some parents have. Indeed, some studies show parents can indeed be concerned about transparency in pediatric AI and its consequences for decision-making (Sisk et al. 2020). Thus, while medical personnel may feel that AI is just “another computer system,” parents may feel differently.

As well as wanting to know the grounds of a diagnosis following identification of ultrasound abnormalities, parents may want to know why incidental findings were among the outputs of the AI, if that is part of its remit. For example, the AI is likely to be programmed to assess aspects, such as the likely pathogenicity of the variant, the potential for some kind of action to be taken, or the age the condition is likely to begin exhibiting symptoms. Yet, parents might be wondering whether the AI is designed to only take into account potential benefits of early disclosure of incidental findings for the fetus or is also designed to take into account the potential benefit for the parents in having this information, such as for their own health. They might want to know if there is some early intervention that could ameliorate the unrelated condition. While the possibility and implications of returning incidental finds should be discussed before an ultrasound is conducted, in some cases, these questions will only occur to parents after a result has been returned.

Respect for autonomy requires obtaining informed consent for medical interventions and tests, which means that patients or parents need to understand its risks and benefits. Informed consent for AI employment may require explanation of unfamiliar or somewhat controversial technologies that cause anxiety in some people, and careful dialogue with parents about a diagnostic system’s strengths and weaknesses (e.g., from limited interpretability). However, it may be more difficult to obtain truly informed consent from patients if we cannot be sure which results will be identified and returned. Although obtaining informed consent is an issue with genomic sequencing in general, the problem is exacerbated by AI if we do not know how decisions about what to report are being made. AI systems should not be designed to bypass parents’ autonomous wishes not to receive incidental findings if they opt out during the consent process.

As the principle of nonmaleficence requires, medical AI must be carefully evaluated against its known harms and its potential risks to human beings (Dias and Torkamani 2019, p. 8). ML models can suffer from generalizability problems when applied to new data, resulting in false positives or negatives. Training and test data, for example, may differ in important but unforeseen ways from data encountered in clinical applications, causing problems known as algorithmic underfitting or overfitting. For example, an overfitted ML model may internalize “noisy” parts of the dataset (Eche et al. 2021) and thus fail to generalize well to other datasets from current children or fetuses. Also, an algorithm that is continuously updated with new data (rather than “locked” after initial training and testing) runs some risk of losing accuracy and thus posing risks of harm to its target population. Hence, it will require ongoing testing and validation.

It is already known that additional risks arise from genetic screening for early illness or abnormality at scale. AI could play a role in scaling up risks. Research shows, for example, that AI can increase overdiagnosis (Capurro et al. 2022). AI may identify many additional variants that are associated with genetic disease but that lack a definitive causative effect or that may not be associated with clinical benefit. This could cause unnecessary anxiety and distress for parents. Consider the detection of variants in the fetus that predispose adults to develop breast cancer when a majority will either never develop tumors or develop them at a life stage where treatment is not beneficial. Prenatal detection could mean that parents might elect to terminate their pregnancy based on a dubious cancer risk.

Another important ethical issue concerns privacy and security of data within AI systems that are trained on copious sensitive data. Analysis of data from current prenatal cases will be most effective when there are large amounts of data from previous cases to compare with, provided the information about the clinical picture of the fetus (e.g., what the fetal abnormalities are) is linked to the genomic data. This becomes important for ultra-rare conditions where individuals are particularly identifiable.

It has been argued that we should not disclose certain genetic test results when they are strong risks to a fetal privacy (Botkin 1995). These considerations are especially relevant in the case of ultra-rare conditions. However, genomic data from individuals with ultra-rare conditions will be an especially valuable resource, when linked to clinical information, and would be highly sought after by pharmaceutical and insurance companies. Access to predictive information, such as incidental findings predisposing the child-to-be and (in most cases) one of their parents to an adult-onset condition (e.g., hereditary breast and ovarian cancer), could have major implications for that family’s ability to receive insurance cover in some locations.

### AI in rapid genomics for time-critical pediatric treatment decisions

Each year millions of infants are born with genetic disorders; perhaps 6% of all children enter the world with serious birth defects of genetic or partially genetic origin (Zarocostas 2006). Fortunately, care is improving for critically unwell children due to increased utilization and speed of GS in neonatal and pediatric intensive care units (PICU) (Collins 2019). Some groups can now sequence genomes in days rather than months, meaning clinicians can receive test results by morning rounds of their next shift (Clark et al. 2019; Gorzynski et al. 2022; Kingsmore et al. 2015). The speed record for GS is now just over 5 h (Doxzen 2022).

Rapid GS has most utility for critically ill children (Clark et al. 2019) for whom a diagnosis in the next 24–48 h has led to improved health outcomes, as well as to a more efficient use of medical resources via transfer to end of life care when further treatment is deemed futile. The diagnostic yield of rapid GS in this population is over 50% and, in some cases, it identifies relatively simple treatments that are lifesaving. More commonly, but also beneficially, it reduces the need for painful and invasive diagnostic investigations before children are transferred to end of life care. Several studies from children’s hospitals worldwide also indicate that rapid GS will create healthcare savings (Carey et al. 2020; Farnaes et al. 2018; Goranitis et al. 2022).

As mentioned previously, clinical interpretation of genetic variants is extremely labor-intensive and time-consuming. Recently, a number of algorithms based on machine learning have been developed that help to automate this process, and may help improve the speed and reduce the cost of GS (De La Vega et al. 2021). However, this development raises the question of how much influence over treatment decisions AI should have. In an acute care setting, variant calling can make the difference between a child’s care continuing or being withdrawn. If variant calling is in the hands of AI systems, then these systems will strongly influence whether some children are offered treatment or instead directed to palliative care. This potentially raises several ethical concerns.

One potential concern is the lack of interpretability of deep learning models. Low algorithmic transparency can hamper trust in recommendations and cause either unjustified uptake or unjustified rejection of AI (Jacovi et al. 2021). The former could cause harm to patients, while the latter could deprive them of benefits. Furthermore, variant calling could be made more complex by a lack of transparency about an AI model’s workings due to proprietary secrecy. We may then ask whether in time-critical situations (e.g., in PICU) minimal transparency—especially when the AI prediction (e.g., diagnosis) or recommendation (e.g., withdrawal of care) is surprising or unexpected—will hinder effective delivery of treatment such that the harm done to children is immediate and potentially irreversible.

Another concern involves misleading AI outputs. This can occur when, for example, algorithms are (perhaps inadvertently) trained on some non-representative data. It is a potential risk to equity or justice as well as nonmaleficence if an AI has been trained on, say, primarily white populations and the infant has non-white genetic ancestry. Furthermore, patients who are statistical outliers can still be classed as part of a larger cluster or segment of cases, and thus be misdiagnosed. ML models cannot recognize this, but expert decision makers (sometimes) can.

Moreover, many or most AI systems, even when validated in the laboratory, have not been extensively tested in real-world situations (Rogers et al. 2021). In variant calling, there is the potential for systems to flag variants that are associated with disease in one situation but not in another. This is particularly worrying for critically ill children. While automation can sometimes minimize human errors in medicine, the aforementioned problem of automation bias (Goddard et al. 2012) suggests a need to provide even stronger evidence that these systems do not harm patients/parents, especially when they are relatively unfamiliar to practitioners. Note that these problems of unrepresentative data and errors in real-life applications are problems that also affect the genomic prenatal (previous section) and reanalysis (next section) settings.

A final concern involves accountability. As noted, AI’s reliability can be hard to establish. An AI system might be accurate for a given set of cases but unreliable or biased for another. And, once again, AI can occasionally make false predictions no competent human would make. Yet because it can be difficult to determine when a deep learning AI model succeeds and fails, it is harder to assign responsibility for patient harms. A responsibility gap (Santoni de Sio and Mecacci 2021) may emerge when there is a failure to clearly assign liability amongst practitioners, tech companies, hospitals, and health systems.

Imagine a child has care withdrawn because of an AI-generated variant call, which turns out to be wrong. Who bears responsibility? Given that such time-critical decisions may have enormous and immediate consequences for very sick children, the responsibility gap problem could be acute. Thus, AI in time-critical pediatric medicine (and other settings) requires careful formulation of accountability mechanisms that assign responsibility fairly to one or (usually) more parties without undermining trust in beneficial AI systems.

### AI for genomic reanalysis in pediatric contexts

Although GS substantially increases diagnostic yield for many genetic conditions, there are many pediatric patients for whom a genetic cause remains unknown. Often this is because knowledge of the genes that cause some diseases and the relevant types of changes in the DNA within known genes is lacking. Some believe that “[i]deally, all unsolved cases would be reanalyzed automatically periodically, and a subset with high likelihood of new findings would be prioritized for manual review” (De La Vega et al. 2021, p. 15). A literature review of 27 reanalysis studies reported a median new diagnosis rate of 15% (0.08–83.33) after one-off reanalysis at a median timeframe of 22 months (Tan et al. 2020). As more variants enter variant databases, many currently undiagnosed cases will probably be solved. This requires that previously analyzed samples from children be reanalyzed. Some institutions are doing this, with increases in diagnostic yield being demonstrated (Dai et al. 2022). Yet this process usually still necessitates considerable manual curation, requiring laboratory scientists to compile and assess new evidence relating to each potentially causative variant, which requires resources.

Typically, the cost of a GS test does not include funding for further analysis down the track. Therefore, if reanalysis was to take place using current methods, someone would need to pay for it. Other questions concern how and when reanalysis should occur: Should it be triggered by the referring clinician or the laboratory? If the clinician, how do we ensure all patients have access to it? If the lab, how often is appropriate to balance benefit versus costs? It is unsurprising, then, that routine automation of reanalysis is considered ideal (Lu et al. 2020).

As in other scenarios, automation of reanalysis using AI could increase the accuracy of variant detection and curation through better incorporation of evidence from databases and improved pattern matching. However, a main advantage would be to increase the *scale* of reanalysis, resulting in more diagnoses for patients. The reduced need for manual curation would reduce human workload, allowing more frequent reanalysis. In fact, reanalysis could be continuous without requiring a trigger from the elapse of a particular time since the last reanalysis or clinician referral. This means that any updates to the bioinformatics pipeline that are implemented (either a new gene identified relating to a particular condition or new information that strengthens or weakens evidence of an association between a variant and a phenotype) would be immediately applied to existing datasets, reducing delays in returning potentially clinically actionable diagnoses to patients.

There are several issues here when AI is involved. An important one is the potential for biases in AI analysis and any consequent injustice and harm against certain individuals or groups. Harmful and unjust bias can result from poorly representative or skewed training data that concerns (say) minoritized groups. As noted, such bias can be more difficult to identify and correct in less transparent “blackbox” systems. Approximately 70% of the existing genome-wide association studies are based on populations with European ancestry (Landry et al. 2018). Hence, there are already inequities in the healthcare provided to individuals of non-European origin when GS is involved. Variants that might very well be common in these populations may be classified as rare and potentially disease-causing in individuals purely because we lack enough population-specific GS data to compare it to (Viswanathan et al. 2018).

Although there is a push internationally to increase the diversity of genomes in these databases, this will take time. If ML is implemented here too early, it may exacerbate existing biases and inequities. Of course, automation of reanalysis without ML would still be beneficial, and reanalysis algorithms could be written and controlled by laboratory scientists as a first step. However, because the progressive introduction of ML is almost inevitable, these issues will need to be carefully considered beforehand.

As with the prenatal scenario, automation of reanalysis using AI in the pediatric context also poses issues for autonomy and consent. Indeed, additional challenges stem from the ongoing nature of the reanalysis: over time the undiagnosed child may reach an age where they have capacity to consent to their own medical care. Yet there may or may not be systems in place to recontact the family to ask whether the now-adult person wishes their data to be indefinitely reanalyzed. One solution could be that the system, as well as reanalyzing the data, also detects when the child is approaching adulthood and notifies the family of the ability to reconsent/withdraw from reanalysis. While this system would reduce the need for genetic health professionals to recontact families, it raises several problems.

First, ideally, rather than just asking for the patient to reconsent when they reach the age of majority, there should be an ongoing conversation from the time when the child is able to comprehend—at an age-appropriate level—that a cause for their condition is being investigated (Jeremic et al. 2016). Second, typically, the medical records will only have contact details for the parents rather than the child. Alerting the parents could violate the child’s right to privacy.

Third, it will sometimes be unclear at the time genomic data is generated whether that child will ever acquire the capacity to consent to this process, and it may be upsetting for parents to receive such a request when their child reaches 18 years of age. Some children may also die before their 18th birthday. Having an automated AI system without some way of assessing whether recontact is appropriate may cause unnecessary distress for families. These issues clearly generate ethical, logistical, and governance challenges for ensuring that the interests and rights of patients and parents are protected.

## Discussion and recommendations

We have shown how genomic AI in prenatal and pediatric settings could lead to a range of positive and negative implications for children and parents and create a number of ethical issues. In our view, the capacity of AI to significantly improve patient care in prenatal genomic testing, time-critical pediatric genomic medicine, and genomic reanalysis domains warrants further research. If sufficiently robust validation and real-world studies find that certain AI tools have benefits that outweigh their drawbacks, it may even be warranted to routinely implement them. As we have seen, genomic AI could generate novel insights and enhance the speed, efficient routinization, scalability, and accuracy of analysis practices. However, key concerns must be addressed to ensure the ethical use of this rapidly developing technology.

Concerns include possibilities of harm, insufficient benefit, and unfair bias. There will likely be greater justified trust on the part of doctors and patients/parents if the AI system has been rigorously tested in both “test” settings and actual clinical settings and shown to be mostly free (or as free as possible) from the potential for causing harm and creating genomic discrimination and inequity. Misguided trust or distrust in AI (Jacovi et al. 2021) may harm children and parents, so clearly establishing the risks, benefits, and fairness of each specific genomic application and model is crucial.

Justified trust will likely be increased if genomic AI tools are more transparent. Therefore, we suggest that interpretable systems should be preferred in these early life settings unless non-transparent or commercial-in-confidence systems are provably more accurate and fairer. Additionally, transparent AI systems could provide more information for autonomous decision making in parents and, as they mature, in children, assuming such information is something they desire.

Until there is wider professional acceptance based on greater justified trust in genomic AI tools in prenatal testing, diagnosing critically ill children, and reanalysis, the responsibility to prevent harm to patients and families from misdiagnosis should be held by medical service decision-makers. That may change as real-world testing of clinical outcomes and warranted faith in the benefits, reliability, and fairness of the software increases. Indeed, if the performance of AI in these settings becomes sufficiently high and robust, it may become unfair to hold the practitioners or hospitals who choose to use the systems wholly accountable for placing their trust in a technology that will never be perfectly free from error (Prictor 2022).

For the foreseeable future (and perhaps always), qualified practitioners should be substantially *involved* in genomic AI applications in these three early life settings. The stakes in genomic medicine are high for present or future children and their parents. Even very accurate machines can make errors, including some errors no human would make, and can in doing so sometimes damage the wellbeing and autonomy of patients and/or their guardians. Therefore, AI should be regarded as a decision support tool. This position would align with WHO’s medical AI guideline that “humans should remain in full control of health-care systems and medical decisions” (World Health Organization 2021, p. 25). Because AI tools lack human understanding, relevant medical decision-makers should oversee and assume accountability for integrating genomic AI in prenatal and pediatric settings.

## Conclusion

Use of AI in genomic medicine presents significant opportunities. AI can potentially analyze massive genomic data sets rapidly, identify novel genetic associations, and make genomic data easier to handle. However, AI presents some risks in early genomics contexts. Genomics is a highly complex area of medicine where uncertainty and variable outcomes are the norm. This can obviously make genomic diagnoses, particularly surprising ones, difficult for some to trust. Such difficulties could be amplified by less transparent AI systems. Using AI in genomics may in some respects make the field generally harder to understand and rely upon. Concerns also arise regarding ending pregnancies without sufficient grounds and not respecting patient autonomy once young humans approach or reach maturity.

One way to promote justifiable AI in prenatal and pediatric is to play close attention to the four well-known ethical notions of non-maleficence, beneficence, justice, and respect for autonomy as well as additional concepts of transparency, accountability, privacy, and trust. We suggested that AI-based recommendations should never entirely substitute for human judgements. Until AI is judged to be extremely reliable, accountability for the deployment of AI tools should tend to fall on practitioners or (more likely) on institutions like hospitals and clinics who make the call about adopting certain algorithms. Sufficient steps should be taken to protect sensitive genomic and other data use in AI systems. Finally, there is a case for saying that not only rigorously tested but *transparent* AI should be preferred where possible to reduce risks of unnoticed error, harm, and bias, and so to increase trust in genomic AI tools.

## Data Availability

Data sharing is not applicable to this article as no datasets were generated or analyzed during the current study.

## Footnotes

1 We exclude AI for embryos (Afnan et al. 2021) from our analysis and focus on the fetal period and early childhood.

2 Although we note that AI could sometimes be expensive.

3 Thanks to a reviewer for pressing these points.

## Declarations

This article does not contain any studies with human or animal subjects performed by any of the authors.
